# Low-dose radiotherapy promotes the formation of tertiary lymphoid structures in lung adenocarcinoma

**DOI:** 10.3389/fimmu.2023.1334408

**Published:** 2024-01-08

**Authors:** Duo Wang, Liuying Huang, Danqi Qian, Yulin Cao, Xiaohan Wu, Peiwen Xu, Liang Ming, Junhui Tang, Zhaohui Huang, Yuan Yin, Leyuan Zhou

**Affiliations:** ^1^ Wuxi Cancer Institute, Affiliated Hospital of Jiangnan University, Wuxi, China; ^2^ Department of Radiation Oncology, Affiliated Hospital of Jiangnan University, Wuxi, China; ^3^ Department of Radiation Oncology, Dushu Lake Hospital Affiliated to Soochow University, Suzhou, China; ^4^ State Key Laboratory of Radiation Medicine and Protection, Soochow University, Suzhou, China

**Keywords:** lung cancer, low dose radiotherapy, tertiary lymphoid structures, immunotherapy, tumor microenvironment

## Abstract

**Purpose:**

A tertiary lymphoid structure (TLS) refers to an organized infiltration of immune cells that is linked to a positive prognosis and improved response to immunotherapy. However, methods that promote TLS formation are limited and challenging to implement in clinical settings. In this study, we aimed to promote the formation and maturation of TLSs in lung adenocarcinoma (LUAD) by combining low-dose radiotherapy (LDRT) with immunotherapy.

**Methods:**

Tissue sections from 198 patients who had undergone surgery were examined. Risk factors for patient survival were assessed, and the relationship between TLSs and five-year survival was analyzed. The Kras-LSL-G12D spontaneous lung cancer mouse model was used to screen the optimal irradiation dose (0/1/2 Gy whole lung irradiation) for promoting TLS formation. LDRT combined with anti-PD-1 was used to promote the formation and maturation of TLSs.

**Results:**

TLS+, TLS^High^, TLS+GC+ and CD8^High^ within TLS+ were associated with a favorable prognosis. LDRT increased the formation of early TLSs in the Kras-LSL-G12D lung cancer mouse model. In addition, LDRT combined with anti-PD-1 treatment can significantly improve the maturity of TLSs in mouse LUAD, resulting in greater antitumor effects. This antitumor effect was strongly associated with the number of CD8+ T cells within the TLSs.

**Conclusion:**

We successfully applied LDRT combined with PD-1 inhibitor therapy for the first time, which increased both the quantity and maturity of TLSs in lung cancer. This approach achieved a promising antitumor effect.

## Introduction

Non-small cell lung cancer (NSCLC) is the most prevalent type of cancer and the leading cause of cancer-related death worldwide (1). NSCLC encompasses squamous cell carcinoma, adenocarcinoma, and large cell carcinoma. Among these subtypes, lung adenocarcinoma (LUAD) is the most common subtype of NSCLC (2). The utilization of innovative screening techniques has enhanced the early diagnosis of NSCLC. Nevertheless, a substantial number of patients experience relapse following treatment, with only a minority attaining sustained disease-free survival (DFS) in the long term (3). Despite certain advancements in radiotherapy, targeted therapy, and immunotherapy for NSCLC, there has been only a marginal improvement in the overall survival rate (4, 5). Consequently, it is imperative to explore novel therapeutic approaches for lung cancer. Recently, tertiary lymphoid structures (TLSs) have garnered attention as an important element associated with the clinical response to immunotherapy (6).

TLSs are lymphoid formations that develop in nonlymphoid tissues during chronic inflammation, such as chronic infection, autoimmune disease, and cancer (7). TLSs are characterized by their vascularization and consist of T-cell regions containing mature dendritic cells, germinal centers with follicular dendritic cells and proliferating B cells, and high endothelial venules (HEV/PNAd) (8). TLSs have been linked to a positive prognosis in various types of cancer, including lung cancer (9), colorectal cancer (10), melanoma (11), and pancreatic cancer (12). Recently, numerous reports have shown that TLSs can enhance the response to immune checkpoint inhibitors (ICIs) treatment (13, 14). The induction of TLS formation for antitumor purposes is a promising area of research. However, current methods are still limited. The main methods include stimulating TLS formation in tumors by injecting T follicular helper (TFH) cells (15), inducing spontaneous TLS formation in the kidneys, liver, and lungs through the conditional deletion of Notch signaling in adult mice using the standard Notch signaling factor Rbpj (16), and inducing TLS formation through the intratumoral injection of the B lymphocyte chemokine CXCL13 (17). However, these methods are challenging to implement in a clinical setting. In recent years, low-dose radiotherapy (LDRT) with doses ranging up to 2 Gy per segment has emerged as a promising approach to enhancing antitumor immunity (18).

In cancer immunotherapy, investigations on the potential of LDRT remain relatively limited. Several studies have provided evidence that LDRT can elicit distinct biological responses in comparison to those of high and moderate doses of radiation (19, 20). Research has demonstrated that LDRT has the potential to augment immune function, impede tumor growth, and bolster the body’s immune response toward malignancies (21). The mechanism by which LDRT enhances immune function primarily involves the activation of various cellular reactions within immune organs and different T-cell signaling pathways (22, 23). Recent clinical data suggest that exposure to low-dose radiation can reprogram the tumor microenvironment (TME). It is also believed that this radiation exposure promotes the infiltration of immune cells, thereby enhancing the antitumor effects (24, 25).

In recent years, there has been an increase in the number of clinical investigations and foundational trials focusing on the combination of radiation therapy and immunotherapy (26–29). However, most studies on this combined therapy primarily concentrate on a specific type of immune cell in the TME, particularly CD8+ T cells (30–32). Importantly, the antitumor effect of the TME is exerted as a whole rather than being attributed to individual cells within it. The TME includes a lymphocyte cluster called the TLS, which consists of various immune cells and operates as a cohesive unit (6). By administering whole-lung LDRT to the Kras-LSL-G12D lung cancer mouse model, we observed an increase in the number of TLSs in the cancer tissues compared with control tumors. Encouragingly, we revealed that LDRT significantly promoted the antitumor effects of anti-PD-1 therapy, offering a cost-effective and straightforward treatment strategy for the future clinical management of NSCLC, particularly in advanced patients.

## Materials and methods

### Patient cohort

This study included a total of 198 patients diagnosed with stage I-III lung adenocarcinoma who had undergone standard surgical resection at the Affiliated Hospital of Jiangnan University between 2016 and 2018. Formalin-fixed and paraffin-embedded specimens were collected from these 198 patients. The researchers conducted a retrospective analysis to evaluate clinicopathological features and five-year survival rates. The assessed clinicopathological characteristics included variables such as age, sex, clinical stage, histological differentiation grade, pleural invasion, vascular invasion, lymphatic invasion, and perineural invasion. Samples were independently evaluated by two experienced pathologists. TNM staging was performed on patients with non-small cell lung cancer according to the 8th edition of the TNM staging system. The study included 87 males and 111 females, with a median age of 64 years (range: 32-82 years). Survival time was measured from the day of surgery until the fifth-year post-surgery.

### Hematoxylin and eosin staining

The tissue sections were deparaffinized in a xylene solution. Then, the sections were hydrated using a high to low concentration gradient of alcohol. Afterwards, the sections were stained with hematoxylin. Subsequently, the sections were washed and placed in alcohol hydrochloride to return to blue, and differentiated in a differentiation solution. Following this, the sections were rinsed and dehydrated using a low to high concentration gradient of alcohol. Next, the sections were stained with alcohol eosin. The slices were then dehydrated in pure alcohol and made transparent in xylene. Finally, after drying, the slices were sealed with neutral resin. All the aforementioned steps were completed using the Leica ST5020 multi-function dyeing machine (Leica, Germany).

### Immunohistochemical and multiplex immunofluorescence staining

Tumor tissues were fixed in 10% formalin, embedded in paraffin, and continuously sectioned. For both staining protocols, 4 μm thick sections were cut from formalin-fixed paraffin-embedded lung cancer tissue. The sections were dewaxed, rehydrated, and then subjected to antigen retrieval by boiling at 97°C for 20 minutes in sodium citrate (pH = 6.0) or Tris-EDTA (pH = 9.0). The endogenous peroxidases were then blocked by incubating the sections in an endogenous peroxidase blocking solution for 10 minutes. Next, the sections were incubated with 10% goat serum at room temperature for 30 minutes to block non-specific binding.

For IHC staining, the tissues were diluted with a monoclonal diluent and then incubated with a monoclonal antibody overnight at 4°C. Antibodies for staining TLS and other related markers in human tissue included CD3 (ab16669, Abcam), CD20 (ab78237, Abcam), CD21 (abs145284, Absin), CD23 (ab92495, Abcam), PNAd (ab111710, Abcam), CD4 (IR379, ABP), and CD8 (IR024, ABP). Antibodies against TLS and T-cell markers for staining mouse tissue included CD3 (ab16669, Abcam), CD19 (ab245235, Abcam), B220 (RA3-6B2, eBioscience), CD21 (abs145284, ABS145284), Absin), CXCL13 (ab78237, Abcam), GL7 (14-5902-82, eBioscience), CD4 (ab183685, Abcam), and CD8 (ab209775, Abcam). For MIF staining, only one antigen was detected per round, including primary antibody incubation, secondary antibody incubation, tyramine signaling amplification (TSA) visualization, and then labeling the next protein after antigen repair and protein blocking with the same antibodies as those used in IHC staining. TSA visualization was achieved using the XTSA 7-color multifactor IHC kit (AXT37025031, Alpha X), which contains the fluorophores (4’,6-diaminidine 2-phenylindole (DAPI), XTSA 480 (CD23), XTSA 520 (CD20 and B220), XTSA 570 (CD3), XTSA 620 (CD21), XTSA 690 (PNAd and CD19), XTSA 780 (CD163 and CXCL13), XTSA signal amplifying solution, and anti-quenching sealing tablets. After labeling all of the antigens in each panel, microwave treatment was performed to remove the TSA antibody complex with EDTA buffer at 97°C for 20 minutes. All slides were redyed with DAPI for 5 minutes and sealed in antifade mounting medium.

### Assessment of TLSs in lung cancer

To evaluate TLSs in patient tumor samples, we implemented the optimization techniques employed in previous investigations (33). TLS^-^positive (TLS^+^) immunocyte clusters were identified using H&E staining and IHC CD20 and CD3 staining. Immunocyte clusters that were not CD20^+^ and CD3^+^ were considered TLS negative (TLS^−^). To quantify TLS density, we quantified the number of TLSs in each slide and divided the slides into TLS^High or^ TLS^Low^ groups by the slide area using 0.08/mm2 as a cutoff value. SlideViewer software (3DHISTECH, Hungary) was utilized to calculate the slide area. CD23 was utilized as a marker for germinal centers, and the statistical methodology remained consistent with what was previously mentioned. The various stages of TLS assessment remain identical to those previously described. TLSs lacking follicular dendritic cells (FDCs) represented the initial phase of TLS development and were denoted as early TLSs (E-TLSs). TLSs containing FDCs but lacking germinal centers (GCs) were referred to as primary follicle-like TLSs (PFL-TLSs), while TLSs with GCs were referred to as secondary follicle-like TLSs (SFL-TLSs). T assess CD8 expression in TLSs, the overall number of germinal centers and CD8-expressing cells within every TLS in each section was obtained. Subsequently, the sum of the CD8/TLS ratios was used as the final metric, wherein a score above 13 denoted CD8^High^ and a scorebelow 13 denoted CD8^Low^.

In the analysis of mouse tumor samples, based on previous research (17), the detection of immune cell aggregation was observed through two staining methods, namely, H&E staining and IHC B220 or CD19 staining. Immune cell aggregation served as an indicator of TLSs (TLS^+^), while the absence of such aggregation indicated a lack of TLSs (TLS^−^). To evaluate the density of TLSs, the number of TLSs per section was calculated and then divided by the area of the section. The assessment of CD3 involved counting the number of T cells (CD3^+^) in the vicinity and within all TLSs. GCs were identified by using GL7 as a marker. The process of dividing the TLS stages and evaluating CD8 positive staining followed the same approach as previously explained. As a validation technique, the spleen of the mice was utilized as a positive control for all the aforementioned staining techniques (**Figure S1A**).

### Tumor models

C57BL/6JSmoc-Kras^em4(LSL-G12D)Smoc^ mice were purchased from Shanghai Model Organisms Center, Inc., (Shanghai, China). C57BL/6J mice were purchased from GemPharmatech (Nanjing, China) and raised under specific pathogen-free conditions. Two spontaneous models of lung cancer were constructed in situ: 1). AAV2/9-CMV bGI-Cre-EGFP-pA (Shanghai Model Organisms Center, Inc., Shanghai, China) was diluted in PBS to make a 2*10^11^/50 µl working fluid. C57BL/6JSmoc-Kras^em4(LSL-G12D)Smoc^ were anesthetized by isoflurane inhalation, and the working solution was given to the lungs of the mice through the nasal cavity, 50 µl/tablet. After eight weeks, lung tumor formation was observed using the Micro-CT imaging system (Quantum GX2, PerkinElmer). 2). C57BL/6J mice were intraperitoneally injected with 800 mg/kg ethyl carbamate (EC) (Sigma, USA) (8% ethyl carbamate prepared with normal saline, 0.1 ml/10 g body weight) twice a week, and lung tumor formation was observed at 20 w after the start of modeling for five consecutive weeks using a micro-CT imaging system of live small animals.

### Tumor therapy

C57BL/6JSmoc-Kras^em4(LSL-G12D)Smoc^ mice were randomly divided into three groups to receive low-dose radiotherapy: (1) control group (unirradiated group), (2) 1 Gy group, and (3) 2 Gy group. Radiation therapy was delivered to the chest. Briefly, mice were anesthetized with isoflurane, and the chest was irradiated with 1 and 2 Gy depending on the experiment using the Biological X-ray irradiometer (RS2000Plus) using 7.2 Gy/minute at 160 KV, 25 mA, 30 cm SSD. Depending on the location, a 3×3 cm collimator was used to focus the radiation. Seven days later, the mice were euthanized, and whole lung samples were collected.

Then, after selecting the optimal irradiation dose, the two models of mice were randomly divided into four groups for combined treatment: (1) control group (untreated group), (2) 1 Gy group, (3) PD-1 inhibitor treatment group (intraperitoneal injection of 10 mg/kg PD-1 inhibitors every three days), and (4) 1 Gy+PD-1 treatment group (radiation and intraperitoneal injection of 10 mg/kg PD-1 inhibitors every three days). Seven days later, the mice were euthanized, and whole lung samples were collected.

### Flow cytometry analyses

Whole lung tissue with tumors was cut into small pieces and then suspended in PBS to form a cell suspension. After combined treatment with collagenase, deoxyribonuccinase I, and hyaluronidase (Sigma, USA), the tumors were digested at 37°C for 50 minutes and subsequently screened through a 70 μm filter (BD Falcon, USA). The immune cells were isolated with a mouse tumor infiltrating tissue monocyte isolation kit (Solar Bio, China). The immune cells were suspended in PBS and stained with specific flow cytometry antibodies after counting (the stained antibodies are listed in **Supplementary Table S1**) and incubated on ice for 30 minutes. After washing with PBS twice, the suspended cells were analyzed by flow cytometry. Flow cytometry data were obtained and analyzed on a NovoCyte Penteon flow cytometer (Agilent, NovoExpress 1.6.2).

### Statistical analyses

We employed RStudio (V3.5.3) and SPSS Statistics 27.0 software to conduct statistical analyses. To determine the TLS density and CD8/TLS thresholds, we utilized receiver operating characteristic (ROC) curve analysis. For categorical variables, we analyzed differences between the groups using the Pearson χ2 test. To assess the relationship between potential risk factors and five-year survival, we used Kaplan−Meier survival curves and Cox proportional hazard regression analyses. To compare the number of pulmonary surface nodules, the number of cells in flow cytometry data, TLS number and density, we employed Student’s t test. We performed other statistical analyses using GraphPad 9.5.1. Statistical significance was determined as a two-tailed p < 0.05 in all tests.

## Results

### The TLS number in LUAD is associated with a favorable prognosis

**Figure 1:**
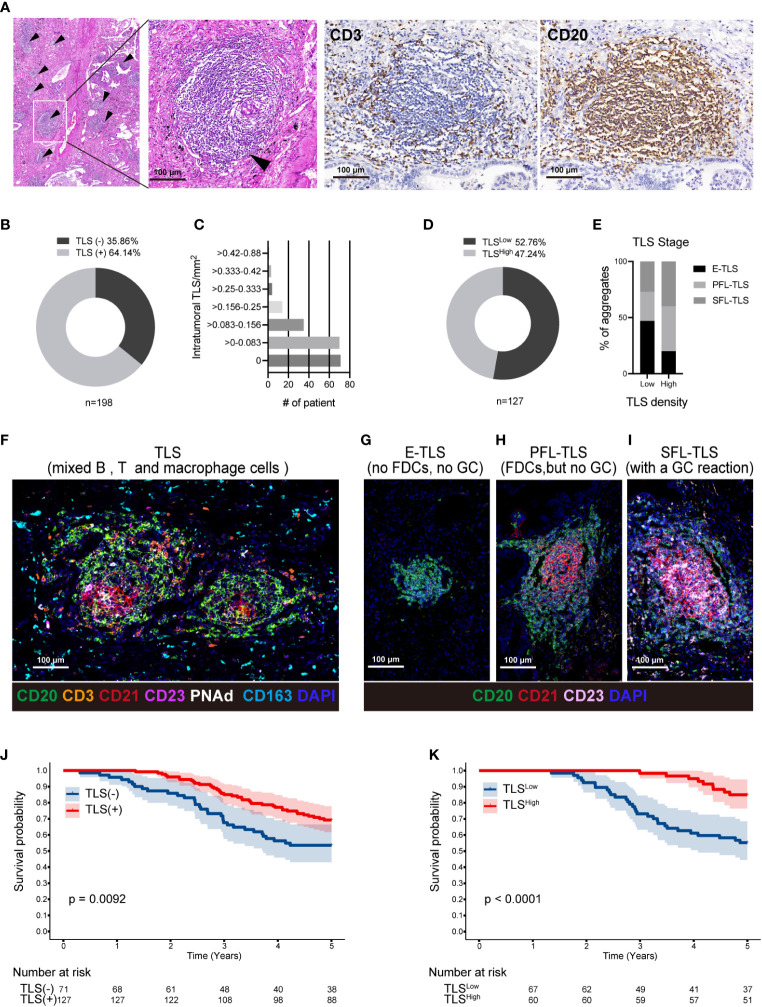
TLSs in LUAD are associated with a favorable prognosis. **(A)** Representative H&E staining of a TLS in LUAD tissue (black arrow); Using the IHC method, CD3 and CD20 were used to locate TLSs in the same region of successive sections. **(B)** Out of a cohort of 198 LUAD patients, 127 had TLS+ and 72 had TLS-. **(C)** The TLS density was divided from low to high into seven density zones, most of which are concentrated in 0-0.083 TLS/mm2. **(D)** Using the ROC curve, TLS+ patients were divided into 67 TLS^Low^ density and 60 TLS^High^ density patients. **(E–I)** T cells (CD3), B cells (CD20), FDCs (CD21), GC cells (CD23), HEVs/PNAd, and macrophages (CD163) were used to define complete TLSs in two LUAD patients with TLS^High^ density using the MIF technique **(F)**. Each TLS was evaluated at a different stage. E-TLS, early TLS; PFL-TLS, primary follicle-like TLS with differentiated FDC (red reticular structure); SFL-TLS, secondary follicle-like TLS with GC reaction (pink area) **(G–I)**. The percentages of E-TLS, PFL-TLS, and SFL-TLS were assessed using the MIF technique in the TLS^Low^ and TLS^High^ groups (E). The image was captured using the ZEISS Axioscan7 full-slice imaging system. **(J)** Five-year Kaplan−Meier survival curves for patients with and without intratumoral TLSs. **(K)**. Five-year Kaplan−Meier survival curves for TLS^Low^ and TLS^High^ patients. The log-rank (Mantel−Cox) test was applied to compare the survival data in Figure **(J, K)**. LUAD, lung adenocarcinoma; MIF, multiple immunofluorescence; IHC, Immunohistochemical techniques; FDC, follicular dendritic cells; GC, germinal center; TLS, tertiary lymphoid structure.

Using primary LUAD tissues from 198 diagnosed LUAD patients at the Affiliated Hospital of Jiangnan University, we conducted a preliminary experiment with H&E staining to investigate the presence of TLSs (**Table 1**). TLSs were defined as dense lymphocyte clusters that positively expressed CD3 and CD20. Among these patients, 127 (64.14%) had TLS structures (**Figures 1A, B**). The TLS densities in the majority of patients were concentrated in the range of 0-0.083 TLS/mm2 (**Figure 1C**). Of these 127 patients, 60 had TLS^High,^ and 67 had TLS^Low^ (**Figure 1D**). We used the MIF assay to evaluate complete TLSs in two patients with high TLS density, including B cells (CD20), follicular dendritic cells (FDCs/CD21), T cells (CD3), GCs (GC/CD23), and surrounding macrophages (CD163) that encircled the cell cluster and contained HEVs (**Figure 1F**). We further divided E-TLSs, PFS-TLSs, and SFL-TLSs by evaluating GCs and FDCs (**Figures 1G–I**). TLSs in the TLS^High^ group exhibited a higher overall maturity than those in the TLS^Low^ group (**Figure 1E**). The relationship between TLS status and clinicopathological features was assessed (**Table 2**), and TLS status was obviously associated with tumor size, TNM stage, pathological grade, lymphatic metastasis and pleural invasion. Among TLS^+^ tumors, TLS density was negatively correlated with clinical stage and positively correlated with the number of infiltrating CD8+ T cells (**Table 3**). In addition, the presence of intratumoral TLSs was linked to a favorable prognosis (HR = 0.55, p = 0.0092) (**Figure 1J**). Furthermore, subgroup analysis showed that improved prognostic outcomes were observed in TLS^+^ patients with different TNM stages, including I (HR = 0.35, p = 0.0031), II (HR = 0.32, p = 0.017), and III (HR = 0.25, p < 0.001) (**Supplementary Figure S2A–C**). Among those TLS^+^ patients, the TLS^High^ group exhibited a better prognosis than the TLS^low^ group (HR = 0.25, p < 0.0001) (**Figure 1K**). Moreover, we evaluated the maturation status of TLSs in the 127 TLS+ patients using CD23 staining and demonstrated that TLS^+^GC^+^ patients (n=63) showed an improved prognosis compared with TLS^+^GC^-^ patients (n=64) (HR=0.44, p=0.013) (**Supplementary Figures S2D, E**). Altogether, these discoveries emphasize the noteworthy impact of intratumoral TLSs on the survival of LUAD patients.

**Table 1:** Clinical and histologic characteristics of the LSCC patient cohort (n = 198) from the Affiliated Hospital of Jiangnan University.

Characteristics	Number (%)	HR^a^ (95% CI)
Gender		0.85
Male	87 (43.9)	0.5-1.5
Female	111 (56.1)	
Age		1.0
<Median	98 (49.5)	0.97-1.0
=Median (64)	17 (8.6)	
>Median	83 (41.9)	
Tumor size		0.97
<Median	91 (46.0)	0.88-1.1
=Median (2 cm)	24 (12.1)	
>Median	83 (41.9)	
TLS (-/+)		0.366*
TLS-	71 (35.9)	0.19-0.70
TLS+	127 (64.1)	
cTNM		1.8*
I	130 (65.8)	1.2-2.7
II	34 (17.1)	
III	34 (17.1)	
Pathological grades (adenocarcinoma)		0.91
Poorly differentiated	64 (32.3)	0.64-1.3
Moderately differentiated	99 (50.0)	
Highly differentiated	35 (17.7)	
Lymphatic metastasis		0.43
Yes	34 (17.1)	0.16-1.1
No	164 (82.9)	
Pleural Invasion		0.40*
Yes	25 (12.6)	0.17-0.90
No	173 (87.4)	
Vascular invasion		1.0
Yes	53 (26.8)	
No	145 (73.2)	
Nerve invasion		0.77
Yes	10 (5.1)	0.30-2.0
No	188 (94.9)	

TNM was assessed according to the 8th Edition of the TNM Staging System for non-small cell lung cancer.

a HR was calculated by univariate Cox regression analysis of Five-year survival, comparing the presence or absence of categorical variables (lymph node metastasis, pleural invasion, vascular invasion, nerve invasion, TLS-/+), or continuous variables (age, tumor size, TNM, pathological grade).

*P < 0.005.

**Table 2:** The TLS (-/+) relationship with the clinical pathological parameters.

Characteristics		patients	TLS		χ^2^	P value
		(n=198)	-	+		
Gender						
	Male	87	26	61	2.408	0.121
	Female	111	45	66		
Age (Median)						
	≤64	115	45	70	1.277	0.258
	>64	83	26	57		
Tumor size (Median)						
	≤2.0	115	49	66	5.435	**0.020**
	>2.0	83	22	61		
cTNM						
	I	130	55	75	7.101	**0.029**
	II	34	9	25		
	III	34	7	27		
Pathological grades (adenocarcinoma)						
	Poorly differentiated	35	27	8	31.697	**<0.001**
	Moderately differentiated	99	28	71		
	Highly differentiated	64	16	48		
Lymphatic metastasis						
	Yes	34	7	27	4.162	**0.041**
	No	164	64	100		
Pleural Invasion						
	Yes	25	7	18	0.768	0.381
	No	173	64	109		
Vascular invasion						
	Yes	53	9	44	11.425	**< 0.001**
	No	145	62	83		
Nerve invasion						
	Yes	10	4	6	0.003	0.954
	No	188	67	121		

The bold values denote statistical significance at P<0.05 level.

**Table 3:** The TLS density relationship with the clinical pathological parameters.

Characteristics		patients	TLS density		χ^2^	P value
		(n=127)	Low	High		
Gender						
	Male	61	34	27	0.419	0.518
	Female	66	33	33		
Age (Median)						
	≤64	70	38	32	0.146	0.702
	>64	57	29	28		
Tumor size (Median)						
	≤2.0	66	38	28	1.281	0.258
	>2.0	61	29	32		
cTNM						
	I	75	42	33	8.560	**0.014**
	II	25	7	18		
	III	27	18	9		
Pathological grades (adenocarcinoma)						
	Poorly differentiated	8	7	1	4.224	0.121
	Moderately differentiated	71	35	36		
	Highly differentiated	48	25	23		
Lymphatic metastasis						
	Yes	27	14	13	0.011	0.916
	No	100	53	47		
Pleural Invasion						
	Yes	18	9	9	0.064	0.800
	No	109	58	51		
Vascular invasion						
	Yes	44	24	20	0.079	0.799
	No	83	43	40		
Nerve invasion						
	Yes	7	4	3	0.001	0.980
	No	120	68	52		
CD8 within TLS						
	Low	73	66	7	97.67	**<0.0001**
	High	54	1	53		

The bold values denote statistical significance at P<0.05 level.

### CD8^+^ T cells in the TLS are associated with a favorable prognosis

**Figure 2:**
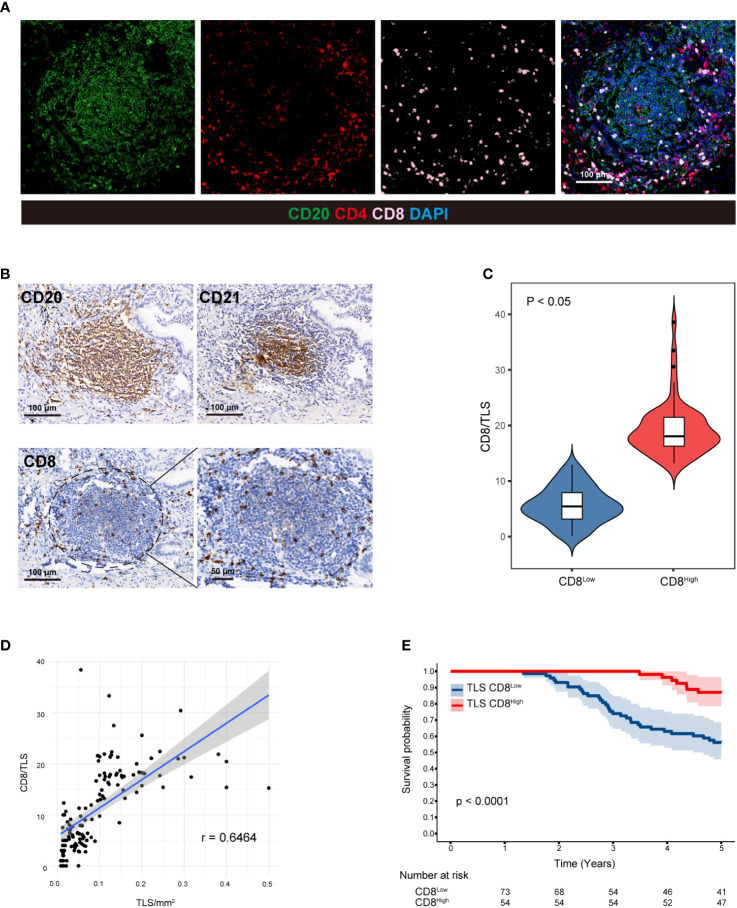
CD8+ T cells in TLSs are associated with a favorable prognosis. **(A)** CD20, CD4, and CD8 were colocalized in two LUAD patients with high-density TLSs using the MIF technique. **(B)** Three consecutive sections of the TLS+ patients were used to evaluate CD20, CD21 and CD8 in the same region using IHC staining, and CD8+ T cells within TLSs were precisely identified. **(C)** CD8/TLS score statistics in high- and low-density TLSs. Student’s t test was used to compare the mean of the two groups. Correlation analysis between **(D)** TLS and CD8/TLS scores. **(E)** Five-year Kaplan−Meier survival curves for CD8^Low^ and CD8^High^ patients. Logarithmic rank (Mantel−Cox) tests were used to compare the survival data in the graphs. MIF, multiple immunofluorescence counting; IHC, Immunohistochemical techniques; TLS, tertiary lymphoid structure.

It has been reported that an abundance CD8^+^ T cells in the TME is associated with a favorable prognosis (34). Therefore, we examined the colocalization of CD20, CD4, and CD8 (**Figure 2A**). CD8+ T cells are an important part of the tumor immune response and play an important role in killing cancer cells, while CD4+ T cells mainly play a role in regulating the tumor immune response (35). As a result, we only evaluated the number of internal CD8^+^ cells in the TLS^+^ group. To accurately locate CD8+ T cells within TLSs, IHC was performed using anti-CD20 and anti-CD21 antibodies on three consecutive sections in the same area (**Figure 2B**). We established CD8/TLS scoring criteria, and based on its ROC curve cutoff (11.29), 57 patients were classified as CD8^High^ and 70 patients as CD8^Low^ (**Figure 2C**). Furthermore, by analyzing the correlation between TLS density and CD8 score (**Table 3**), we observed a positive correlation between CD8/TLS score and TLS density (r=0.79) (**Figure 2D**). Notably, patients with CD8^High^ exhibited a better prognosis than CD8^Low^ patients (HR=0.23, p < 0.0001) (**Figure 2E**). In summary, these findings indicate that in the TLS^+^ group, high-density TLSs predict increased infiltration of CD8+ T cells, which may serve as an important indicator of the survival rate in LUAD patients.

### Comparative analysis of TLSs in Kras-LSL-G12D models and Ethyl Carbamate-induced lung adenocarcinoma mouse models

**Figure 3:**
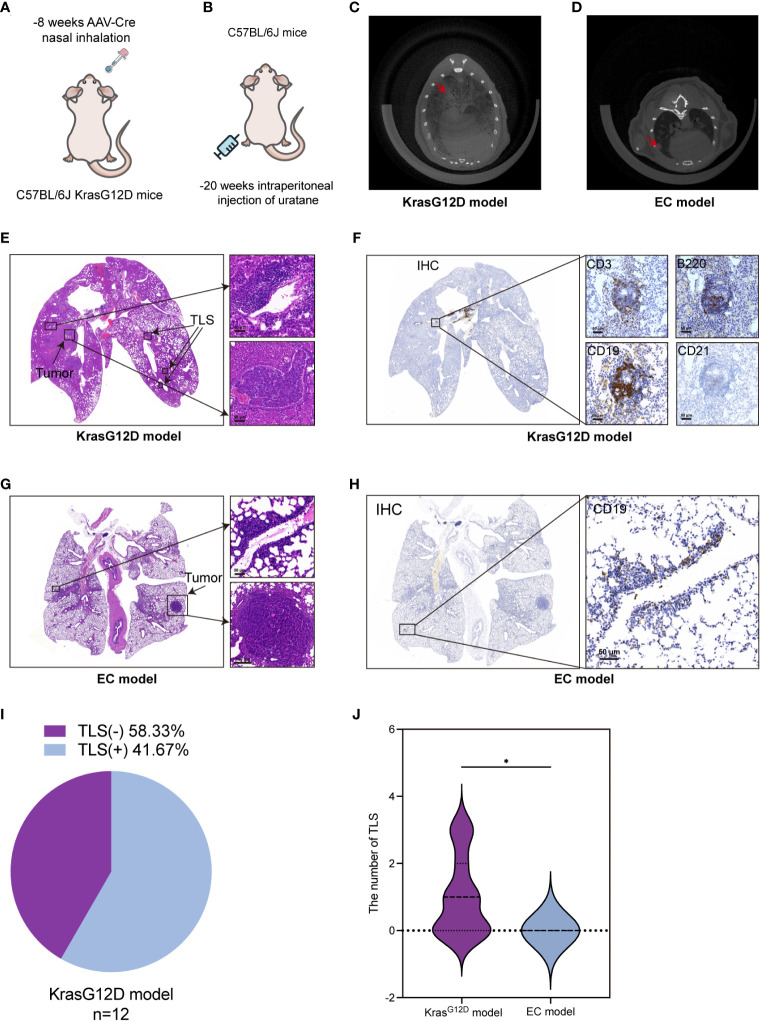
TLSs are present in a mouse lung cancer model. **(A, B)** KrasG12D lung cancer mouse model and EC-induced mouse lung cancer model diagram. **(C, D)** CT images of lung cancer in two mouse models. **(E)** H&E staining of TLSs and tumors in the KrasG12D model. **(F)** TLSs in KrasG12D model mice were evaluated by CD3, B220, CD19, and CD21 at the same location in four consecutive sections using IHC. **(G)** H&E staining of tumor-infiltrating lymphocytes and tumor cells in EC model mice. **(H)** The main marker CD19 was used to determine the presence of TLSs in EC model mice by IHC. **(I)** Ratio of TLS-/+ in the KrasG12D model (n=12). **(J)** Comparison of TLS quantity between the two models. IHC, Immunohistochemical techniques; EC, ethyl carbamate TIL, tumor infiltrating lymphocytes; TLS, tertiary lymphoid structure. *p<0.05.

Based on the aforementioned analyses of the clinical cases, we discovered that the presence of TLSs, higher TLS density, and a more advanced stage of TLS are indicators of a favorable prognosis. Therefore, our aim was to investigate methods that increase the number of TLSs and promote their transition to a more advanced stage through animal experiments. As TLSs are not present in transplanted tumor models or metastatic tumor models (36), we selected a mouse model of spontaneous lung adenocarcinoma. This model included the genetically engineered mouse model (C57BL/6JSmoc-Kras^em4(LSL-G12D)^Smoc, AAV-Cre of LUAD, hereafter referred to as the KrasG12D model) and a more cost-effective ethyl carbamate (EC)-induced lung adenocarcinoma model (EC model) (**Figures 3A, B**) (37). We used CT scans to confirm the presence of lung cancer in mice (**Figures 3C, D**). In spontaneous native KrasG12D mice, TLSs exhibited similar characteristics to human LUAD, making them the gold standard preclinical model for studying this type of cancer. We identified TLSs in KrasG12D mice through the morphology of lymphocyte aggregation using H&E staining and the presence of B220, CD19, and CD3 markers (**Figures 3E, F**). TLSs were present in 58.33% (n = 12) of KrasG12D mice (**Figure 3I**) and were similar to those in human LUAD tumors. TLSs were observed in KrasG12D mouse tumors, but the composition of TLSs in these tumors was mainly B cells with a limited presence of T cells and an incomplete FDC (CD21) network (**Figure 3F**). However, in the EC model, we failed to observe TLSs using H&E staining and CD19, a commonly used marker for TLSs (**Figures 3G, H**). Our analysis revealed that TLSs can be observed in the KrasG12D model but not in the EC model (**Figure 3J**). Therefore, we mainly used the KrasG12D model for subsequent studies.

### LDRT can promote the infiltration of immune cells and the formation of TLSs in a mouse lung cancer model

**Figure 4:**
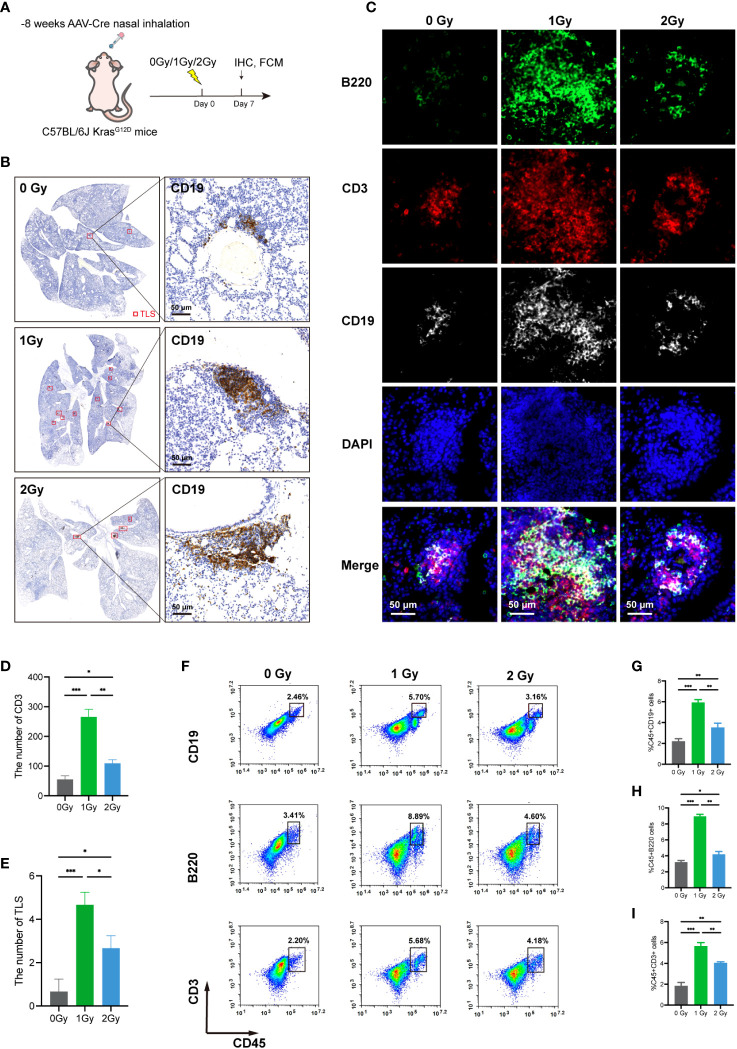
LDRT can promote the infiltration of immune cells and the formation of TLSs in lung cancer model mice. **(A)** Irradiation schedule of KrasG12D model mice. **(B)** CD19 (TLS main marker) IHC staining was used to assess TLS numbers after 0/1/2 Gy irradiation using panoramic scan techniques (n=3 per group). **(C)** MIF staining of B220, CD3, and CD19 in the 0/1/2 Gy group (n=3 per group). The number of CD3+ T cells and the number of TLSs in the **(D, E)**. 0/1/2 Gy group were evaluated, and the mean values of the two groups were compared by Student’s t test. **(F–I)** Flow cytometry analysis of mouse lung mononuclear cell suspensions and the percentages of CD19+ B cells, B220+ B cells, and CD3+ T cells in the corresponding groups (n = 3 per group). Student’s t test was used to compare the mean between groups, and the results are shown as the mean ± SD. *p<0.05, **p<0.01, ***p<0.001. MIF, multiple immunofluorescence counting; IHC, Immunohistochemical techniques; TLS, tertiary lymphoid structure.

The findings of various studies have demonstrated that LDRT possesses the ability to reprogram the TME and facilitate extensive immune cell infiltration (18, 29). We evaluated the effect of whole-lung LDRT on the development of TLSs in mouse lung cancer. The LDRT dose was administered using a gradient irradiation of 0/1/2 Gy. (**Figure 4A**). The infiltration of CD3+ T, B220+ B, and CD19+ B cells in the lung and the number of TLSs formed were assessed seven days after LDRT (**Figures 4B, C**). Interestingly, when compared to the 0 and 2 Gy groups, only 1 Gy irradiation obviously increased TLS production and the number of CD3+ T cells in the tumor tissues (**Figures 4D, E**), which was further confirmed by the flow cytometry results (**Figures 4F–I**). Overall, these findings showed that 1 Gy radiotherapy can enhance the infiltration of immune cells and the formation of TLSs. However, further exploration of combination therapies is warranted, as many of the TLSs observed were immature.

### LDRT combined with anti-PD-1 therapy can promote the formation and maturation of TLSs, enhance the anticancer effect, and increase the number of internal CD8+ T cells

**Figure 5:**
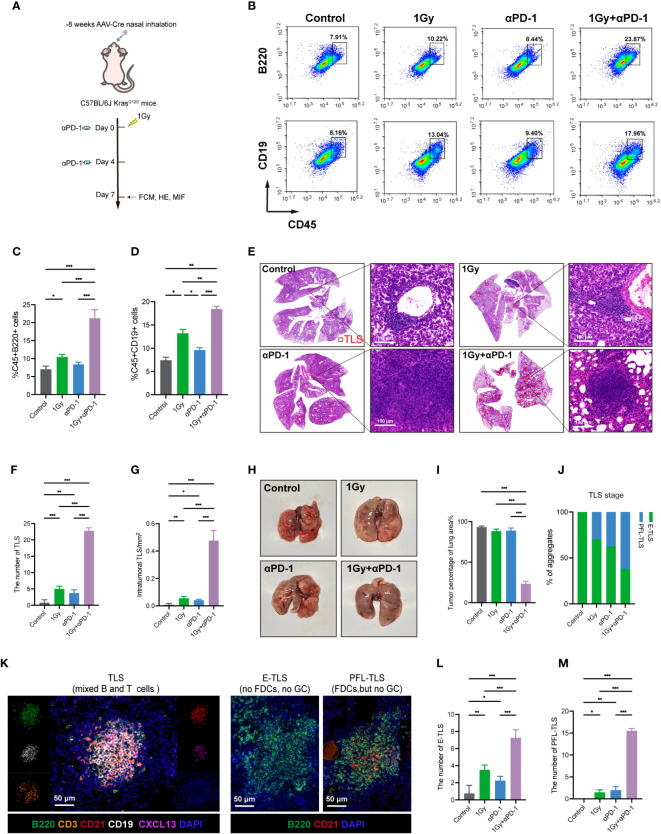
LDRT combined with anti-PD-1 therapy can promote the formation and maturation of TLSs and enhance the anticancer effect. **(A)** KrasG12D model mouse therapy model. **(B–D)** Flow cytometry analysis and percentage of CD19+ B cells and B220+ B cells in mouse whole lung mononuclear cell suspensions (n = 4 per group). Student’s t test was used to compare the mean values between groups, and the results are shown as the mean ± SD. **(E)** TLS representation of H&E staining in different treatment groups. **(F, G)** Statistical graph of the number and density of TLSs in each group (n=4 for each group). Student’s t test was used to compare the mean between the groups, and the results are shown as the mean ± SD. **(H)** Lung surface morphology in different treatment groups. **(I)** Statistical plots of the percentage of lung consolidation area under the total lung in each group according to H&E staining (n=4 for each group). Student’s t test was used to compare the mean value between groups, and the result is shown as the mean ± SD. **(J)** Statistics on the percentage of E-TLS and PFS-TLS for each group. **(K)** The colocalization of T cells (CD3), B cells (B220 and CD19), FDC (CD21) and B lymphocyte chemokine (CXCL13) using MIF technology was used to identify complete TLSs in the different mouse groups. Each TLS was evaluated at a different depth. E-TLS, early TLS; PFL-TLS, primary follicle-like TLS with differentiated FDC (red reticular structure). The image was captured using the ZEISS Axioscan7 imaging system. **(L, M)** Comparison of the number of E-TLSs and PPL-TLSs in each group (n=4 for each group). Student’s t test was used to compare the mean between groups, and the result is shown as the mean ± SD. *p<0.05, **p<0.01, ***p<0.001. MIF, multiple immunofluorescence counting; IHC, Immunohistochemical techniques; TLS, tertiary lymphoid structure.

Previous studies have demonstrated that TLSs can enhance ICI therapeutic reactivity (13, 38). Therefore, we conducted further investigations to explore whether LDRT could enhance the anticancer effect of PD-1 inhibitors. In the KrasG12D model, whole-lung irradiation with 1 Gy was administered, and intraperitoneal injection of anti-PD-1 was given on the day of irradiation and day 4 (dual-treatment group) (**Figure 5A**). First, the flow cytometry analysis showed that the proportions of the two major B-cell types of TLSs, B220+ B cells and CD19+ B cells, were significantly increased in the dual-treatment group. (**Figures 5B–D**). In the subsequent H&E staining, it was observed that LDRT promoted the formation of TLSs, consistent with previous results. Notably, while the anti-PD-1 group showed only a slight increase in TLS numbers, the dual-treatment group exhibited a significant increase in both the number and density of TLSs compared to the LDRT group. (**Figures 5E–G**).

Due to the diffuse growth of lung tumors in the KrasG12D model, they do not exhibit nodular growth. Hence, the assessment of the lung specimen relies solely on its morphology. Our findings indicated that the surface morphology of the lung in the dual-treatment group was noticeably smoother and exhibited significantly better normalization compared to the other groups. (**Figure 5H**). H&E staining revealed a significantly higher proportion of normal lung tissue in the dual-treatment group than in the other groups (**Figures 5E, I**; **Supplementary Figure S3A**). Moreover, we performed MIF staining using B-cell markers (B220, CD19), T-cell markers (CD3), FDC markers (CD21), GC markers (GL7), and B lymphocyte chemokines (CXCL13), and TLS maturation was assessed using B220, CD21 and GL7 (**Figure 5K**). The level of TLS maturity directly correlates with its potential antitumor efficacy, meaning that a higher level of TLS maturity results in a stronger antitumor effect (38). Interestingly, we observed that the dual-treatment group exhibited the highest proportion of TLSs (**Figure 5J**) while also demonstrating the highest proportions of both E-TLSs and PFL-TLSs (**Figures 5L, M**). However, the most mature TLS type (SFL-TLSs), assessed by GC expression, was not observed in our study, with mouse spleens used as positive controls (**Supplementary Figure S1A**). CXCL13, a chemokine specific to B lymphocytes, has the ability to attract B-cell aggregates and facilitate the development and maturation of TLSs to some extent. Interestingly, the presence of CXCL13 was observed only in the dual-treatment group (**Supplementary Figure S3B**), which aligned with the aforementioned results of the dual-treatment group and indicated a higher level of TLS maturity.

**Figure 6:**
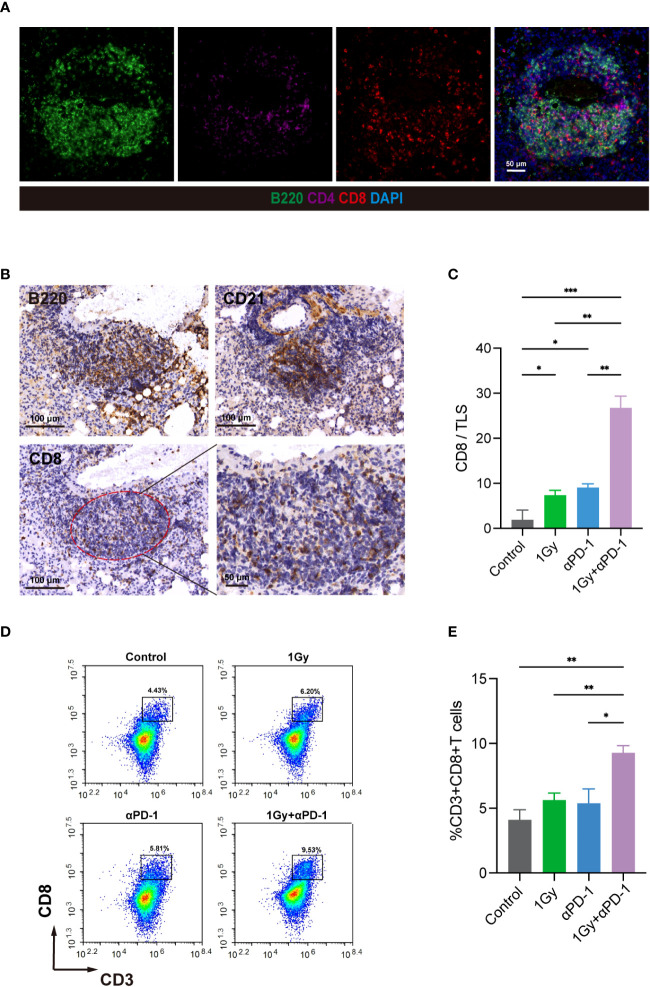
LDRT combined with anti-PD-1 therapy can promote the formation and maturation of TLSs, enhance the anticancer effect, and increase the number of internal CD8+ T cells. **(A)** In the KrasG12D model, MIF technology was used to evaluate the colocalization of B220, CD4 and CD8. **(B)** In three consecutive sections, IHC technology was used to evaluate B220, CD21 and CD8 at the same site and precisely identify CD8+ T cells within TLSs. **(C)** CD8/TLS scores among the groups. Student’s t test was used to compare the mean values between groups, and the results are shown as the mean ± SD. **(D, E)** Flow cytometry analysis of the percentage of CD8+ T cells in mouse whole lung mononuclear cell suspensions (n = 4 per group). Student’s t test was used to compare the mean between the groups, and the results are shown as the mean ± SD. *p<0.05, **p<0.01, ***p<0.001. MIF, multiple immunofluorescence counting; IHC, Immunohistochemical techniques; TLS, tertiary lymphoid structure.

Subsequently, we utilized MIF staining to evaluate the colocalization of B220, CD4, and CD8 in tissue sections from KrasG12D model mice (**Figure 6A**). IHC was then employed to accurately identify CD8+ T cells within the TLS range in three consecutive sections in the same area using anti-B220 and anti-CD21 antibodies (**Figure 6B**). CD8/TLS scoring metrics were also utilized. The CD8/TLS scores in the dual-treatment group were found to be significantly higher than those in the other groups (**Figure 6C**). Flow cytometry analyses further confirmed a significant increase in CD8+ T cell numbers in the dual-treatment group (**Figures 6D, E**). In summary, the aforementioned results demonstrated that LDRT (1 Gy) combined with anti-PD-1 treatment can effectively enhance the generation and maturation of TLSs in the KrasG12D model. Additionally, it promotes the generation of CD8+ T cells within TLSs, thereby enhancing the antitumor effect.

## Discussion

In this study, we investigated the effect of LDRT on TLSs for the first time and revealed that LDRT (1 Gy) enhances the formation of TLSs. Additionally, we found that combining LDRT with anti-PD-1 not only increased the number of TLSs in the tumor but also improved their maturity, resulting in significantly enhanced antitumor effects compared with those of the single treatments.

The TME includes tumor cells as well as nontumor cells from different lineages. The role of the immune microenvironment in the development and progression of tumors has been extensively researched since the successful implementation of immunotherapy for NSCLC (39, 40). TLSs are believed to play a critical role in immune responses, both cellular and humoral, against tumor cells. They have also been found to be positively associated with clinical outcomes in patients with various solid tumors (41–43). Based on this research, we found that the TLS levels in LUAD tumors were indeed strongly associated with better survival. In particular, the presence and density of TLSs, and specifically CD8^+^ T cells within these structures, can serve as a superior prognostic indicator. Furthermore, we conducted a comparison between the presence of TLSs in GC^-^TLSs and GC^+^TLSs. Our findings are consistent with previous studies (10, 33, 44, 45), showing that GC^+^TLSs but not GC^−^TLSs are linked to better survival rates. Our study underscores the powerful antitumor effects of TLSs within primary tumors. However, it is essential to acknowledge that the precision of our data might be impacted by the relatively small sample size. Therefore, further validation of our findings with a larger sample size is necessary.

While there have been documented techniques for inducing the generation of TLSs, their implementation poses a substantial challenge (15–17). The discovery of a more pragmatic approach to facilitate the development and maturation of TLSs in a clinical context is urgently needed. Prior investigations have explored the utilization of LDRT to counteract immune deficiency in the TME and transform “cold tumors” into “hot tumors” (16). In this research, using the KrasG12D model, we illustrated that a dose of 1 Gy results in the heightened infiltration of immune cells within the TME and an increase in TLS formation, suggesting that LDRT may promote the efficiency of tumor immunotherapy by transforming “cold tumors” into “hot tumors”. Previous research has indicated that the response of neoadjuvant immune checkpoint blockade (ICB) treatment in melanoma patients is linked to the number of TLSs in their tumors (14). This finding prompted us to investigate whether LDRT enhances the anticancer effect of ICI treatment by inducing the development of TLSs. Taking these findings and the induction effect of LDRT on the formation of TLSs revealed by our study into consideration, we evaluated the efficacy of 1 Gy whole lung radiotherapy and PD-1 blockade combination therapy in a KrasG12D lung cancer mouse model. Our findings revealed that the combined application of LDRT and PD-1 blockade can augment both the quantity and maturity of intratumoral TLSs, along with a noteworthy increase in the number of CD8+ T cells within TLSs, resulting in excellent anticancer effects. In the context of TLS formation, it has been discovered that the induction of TLS can be attributed to interleukin-17 (IL-17) produced by T cells. IL-17 has the ability to stimulate the expression of CXCL13 and CCL19 in stromal cells of mice, thereby promoting the development of bronchial associated lymphoid tissue (iBALT). This represents a type of TLS formation in lung tissue (6, 41). Our study also observed that the combination of 1Gy radiation and anti-PD-1 treatment could induce the expression of CXCL13, which may play a role in TLS formation. However, the specific mechanism behind TLS formation remains controversial, and it is a key area for future research. In addition, the limited duration of treatment may have been the primary factor contributing to the modest effect observed with monotherapy, particularly with anti-PD-1 therapy. Furthermore, the short treatment duration may explain the absence of SFL-TLSs. Therefore, future experiments with prolonged treatment periods should be performed to address this issue.

To summarize, our research developed an approach to increase the formation and maturation of TLSs in a KrasG12D mouse model, which consequently enhanced the treatment efficacy against tumors. This investigation presents novel concepts for treating patients with advanced-stage lung cancer, offering potential improvements in clinical care. It is crucial to acknowledge that our discoveries rely on observations from a singular animal model, necessitating additional studies to assess the reproducibility of this technique in various tumor models. Moreover, exploring the mechanism by which this specific combination therapy promotes the maturation of TLSs warrants further inquiry.

## Data availability statement

The original contributions presented in the study are included in the article/**Supplementary Material**. Further inquiries can be directed to the corresponding authors.

## Ethics statement

The studies involving humans were approved by the Institutional Review Board of the Affiliated Hospital of Jiangnan University. The studies were conducted in accordance with the local legislation and institutional requirements. The participants provided their written informed consent to participate in this study. The animal study was approved by Institutional Animal Care and Use Committee of Jiangnan University. The study was conducted in accordance with the local legislation and institutional requirements.

## Author contributions

DW: Conceptualization, Data curation, Formal analysis, Investigation, Methodology, Software, Supervision, Validation, Visualization, Writing – original draft, Writing – review & editing. LH: Writing – review & editing. DQ: Writing – review & editing. YC: Writing – review & editing. XW: Writing – review & editing. PX: Writing – review & editing. LM: Writing – review & editing. JT: Writing – review & editing. ZH: Funding acquisition, Resources, Writing – review & editing. YY: Funding acquisition, Writing – review & editing. LZ: Funding acquisition, Project administration, Resources, Writing – review & editing.

## References

[1] Siegel RL Miller KD Fuchs HE Jemal A. Cancer statistics, 2022. CA: A Cancer J Clin (2022) 72(1):7–33. doi: 10.3322/caac.21708 35020204

[2] Thai AA Solomon BJ Sequist LV Gainor JF Heist RS. Lung cancer. Lancet (2021) 398(10299):535–54. doi: 10.1016/s0140-6736(21)00312-3 34273294

[3] Asamura H Chansky K Crowley J Goldstraw P Rusch VW Vansteenkiste JF. The international association for the study of lung cancer lung cancer staging project: proposals for the revision of the N descriptors in the forthcoming 8th edition of the TNM classification for lung cancer. J Thorac Oncol (2015) 10(12):1675–84. doi: 10.1097/jto.0000000000000678 26709477

[4] Sun Y Dai H Chen S Zhang Y Wu T Cao X. Disruption of chromosomal architecture of cox2 locus sensitizes lung cancer cells to radiotherapy. Mol Ther (2018) 26(10):2456–65. doi: 10.1016/j.ymthe.2018.08.002 PMC6171098 30131302

[5] Xu P Xiao H Yang Q Hu R Jiang L Bi R. The USP21/YY1/SNHG16 axis contributes to tumor proliferation, migration, and invasion of non-small-cell lung cancer. Exp Mol Med (2020) 52(1):41–55. doi: 10.1038/s12276-019-0356-6 31956270 PMC7000404

[6] Schumacher TN Thommen DS. Tertiary lymphoid structures in cancer. Science (2022) 375(6576):eabf9419. doi: 10.1126/science.abf9419 34990248

[7] Lauss M Donia M Svane IM Jönsson G. B cells and tertiary lymphoid structures: friends or foes in cancer immunotherapy? Clin Cancer Res (2022) 28(9):1751–8. doi: 10.1158/1078-0432.ccr-21-1130 PMC9306440 34965949

[8] Goc J Fridman W-H Hammond SA Sautès-Fridman C Dieu-Nosjean M-C. Tertiary lymphoid structures in human lung cancers, a new driver of antitumor immune responses. OncoImmunology (2014) 3:e28976. doi: 10.4161/onci.28976 25083325 PMC4106161

[9] Curtis JL. Wouldn’t you like to know: are tertiary lymphoid structures necessary for lung defence? Eur Respir J (2021) 57(4). doi: 10.1183/13993003.04352-2020 33858851

[10] Posch F Silina K Leibl S Mündlein A Moch H Siebenhüner A. Maturation of tertiary lymphoid structures and recurrence of stage II and III colorectal cancer. OncoImmunology (2018) 7(2):e1378844. doi: 10.1080/2162402x.2017.1378844 29416939 PMC5798199

[11] Azimi F Scolyer RA Rumcheva P Moncrieff M Murali R McCarthy SW. Tumor-infiltrating lymphocyte grade is an independent predictor of sentinel lymph node status and survival in patients with cutaneous melanoma. J Clin Oncol (2012) 30(21):2678–83. doi: 10.1200/jco.2011.37.8539 22711850

[12] Hiraoka N Ino Y Yamazaki-Itoh R Kanai Y Kosuge T Shimada K. Intratumoral tertiary lymphoid organ is a favourable prognosticator in patients with pancreatic cancer. Br J Cancer (2022) 112(11):1782–90. doi: 10.1038/bjc.2015.145 PMC4647237 25942397

[13] Helmink BA Reddy SM Gao J Zhang S Basar R Thakur R. B cells and tertiary lymphoid structures promote immunotherapy response. Nature (2020) 577(7791):549–55. doi: 10.1038/s41586-019-1922-8 PMC8762581 31942075

[14] Cabrita R Lauss M Sanna A Donia M Skaarup Larsen M Mitra S. Tertiary lymphoid structures improve immunotherapy and survival in melanoma. Nature (2020) 577(7791):561–5. doi: 10.1038/s41586-019-1914-8 31942071

[15] Chaurio RA Anadon CM Lee Costich T Payne KK Biswas S Harro CM. TGF-β-mediated silencing of genomic organizer SATB1 promotes Tfh cell differentiation and formation of intra-tumoral tertiary lymphoid structures. Immunity (2022) 55(1):115–128 e119. doi: 10.1016/j.immuni.2021.12.007 35021053 PMC8852221

[16] Fleig S Kapanadze T Bernier-Latmani J Lill JK Wyss T Gamrekelashvili J. Loss of vascular endothelial notch signaling promotes spontaneous formation of tertiary lymphoid structures. Nat Commun (2022) 13(1):2022. doi: 10.1038/s41467-022-29701-x 35440634 PMC9018798

[17] Delvecchio FR Fincham REA Spear S Clear A Roy-Luzarraga M Balkwill FR. Pancreatic cancer chemotherapy is potentiated by induction of tertiary lymphoid structures in mice. Cell Mol Gastroenterol Hepatol (2021) 12(5):1543–65. doi: 10.1016/j.jcmgh.2021.06.023 PMC8529396 34252585

[18] Gao L Zhang A. Low-dose radiotherapy effects the progression of anti-tumor response. Trans Oncol (2023) 35:101710. doi: 10.1016/j.tranon.2023.101710 PMC10277580 37320873

[19] Abbassi LM Arsène-Henry A Amessis M Kirova YM. Radiation dose to the low axilla in patients treated for early-stage breast cancer by locoregional intensity-modulated radiotherapy (IMRT). Cancer/Radiothérapie (2021) 26(3):445–9. doi: 10.1016/j.canrad.2021.06.002 34175223

[20] Pei S Chen K Yang Y Chen L Zhu X. A retrospective cohort study of low-dose intensity-modulated radiotherapy for unresectable liver metastases. J Int Med Res (2019) 48(4):300060519892382. doi: 10.1177/0300060519892382 31885298 PMC7607147

[21] Yang G Li W Jiang H Liang X Zhao Y Yu D. Low-dose radiation may be a novel approach to enhance the effectiveness of cancer therapeutics. Int J Cancer (2016) 139(10):2157–68. doi: 10.1002/ijc.30235 27299986

[22] Liu J Zhou J Wu M Hu C Yang J Li D. Low-dose total body irradiation can enhance systemic immune related response induced by hypo-fractionated radiation. Front Immunol (2019) 10:317. doi: 10.3389/fimmu.2019.00317 30873170 PMC6401363

[23] Rödel F Frey B Manda K Hildebrandt G Hehlgans S Keilholz L. Immunomodulatory properties and molecular effects in inflammatory diseases of low-dose x-irradiation. Front Oncol (2012) 2:120. doi: 10.3389/fonc.2012.00120 23057008 PMC3457026

[24] Klug F Prakash H Huber Peter E Seibel T Bender N Halama N. Low-dose irradiation programs macrophage differentiation to an iNOS^+^/M1 phenotype that orchestrates effective T cell immunotherapy. Cancer Cell (2013) 24(5):589–602. doi: 10.1016/j.ccr.2013.09.014 24209604

[25] Barsoumian HB Ramapriyan R Younes AI Caetano MS Menon H Comeaux NI. Low-dose radiation treatment enhances systemic antitumor immune responses by overcoming the inhibitory stroma. J ImmunoTher Cancer (2020) 8(2). doi: 10.1136/jitc-2020-000537 PMC7592253 33106386

[26] He K Patel RR Barsoumian HB Chang JY Tang C Comeaux NI. Phase II trial of high-dose radiotherapy vs. Low-dose radiation, demonstrating low-dose mediated immune-cell infiltration. Int J Radiat Oncol • Biol • Phys (2021) 111(3). doi: 10.1016/j.ijrobp.2021.07.270

[27] Yin L Xue J Li R Zhou L Zhang Y Zhang X. Effect of low-dose radiotherapy on abscopal responses to hypofractionated radiotherapy and anti-PD1 in mice and NSCLC patients. Int J Radiat Oncol • Biol • Phys (2020) 108(3). doi: 10.1016/j.ijrobp.2020.07.1741 32417411

[28] Zhou L Yu M Chen L Zhang Y Jiang Z Liu Y. Marvelous objective response of low dose radiotherapy plus ICIs for extended stage small cell lung cancer. J Clin Oncol (2020) 38(15_suppl):e21097. doi: 10.1200/jco.2020.38.15_suppl.e21097

[29] Herrera FG Ronet C Ochoa de Olza M Barras D Crespo I Andreatta M. Low-dose radiotherapy reverses tumor immune desertification and resistance to immunotherapy. Cancer Discov (2022) 12(1):108–33. doi: 10.1158/2159-8290.cd-21-0003 PMC9401506 34479871

[30] Sanmamed MF Nie X Desai SS Villaroel-Espindola F Badri T Zhao D. A burned-out CD8+ T-cell subset expands in the tumor microenvironment and curbs cancer immunotherapy. Cancer Discov (2021) 11(7):1700–15. doi: 10.1158/2159-8290.cd-20-0962 PMC9421941 33658301

[31] Xie Q Ding J Chen Y. Role of CD8+ T lymphocyte cells: Interplay with stromal cells in tumor microenvironment. Acta Pharm Sin B (2021) 11(6):1365–78. doi: 10.1016/j.apsb.2021.03.027 PMC8245853 34221857

[32] Chen H-y Xu L Li L-f Liu X-x Gao J-x Bai Y-r. Inhibiting the CD8+ T cell infiltration in the tumor microenvironment after radiotherapy is an important mechanism of radioresistance. Sci Rep (2018) 8(1):11934. doi: 10.1038/s41598-018-30417-6 30093664 PMC6085329

[33] Siliņa K Soltermann A Attar FM Casanova R Uckeley ZM Thut H. Germinal centers determine the prognostic relevance of tertiary lymphoid structures and are impaired by corticosteroids in lung squamous cell carcinoma. Cancer Res (2018) 78(5):1308–20. doi: 10.1158/0008-5472.can-17-1987 29279354

[34] Tu MM Lee FYF Jones RT Kimball AK Saravia E Graziano RF. Targeting DDR2 enhances tumor response to anti-PD-1 immunotherapy. Sci Adv (2019) 5(2):eaav2437. doi: 10.1126/sciadv.aav2437 30801016 PMC6382401

[35] Ostroumov D Fekete-Drimusz N Saborowski M Kühnel F Woller N. CD4 and CD8 T lymphocyte interplay in controlling tumor growth. Cell Mol Life Sci (2017) 75(4):689–713. doi: 10.1007/s00018-017-2686-7 29032503 PMC5769828

[36] Joshi NS Akama-Garren EH Lu Y Lee D-Y Chang GP Li A. Regulatory T cells in tumor-associated tertiary lymphoid structures suppress anti-tumor T cell responses. Immunity (2015) 43(3):579–90. doi: 10.1016/j.immuni.2015.08.006 PMC4826619 26341400

[37] Miller YE Dwyer-Nield LD Keith RL Le M Franklin WA Malkinson AM. Induction of a high incidence of lung tumors in C57BL/6 mice with multiple ethyl carbamate injections. Cancer Lett (2003) 198(2):139–44. doi: 10.1016/s0304-3835(03)00309-4 12957351

[38] Wolf HF Maxime M Guilhem P Anne C Isaïas H Catherine S-F. Tertiary lymphoid structures and B cells: An intratumoral immunity cycle. Immunity (2023) 56(10):2254–69. doi: 10.1016/j.immuni.2023.08.009 37699391

[39] Borghaei H Paz-Ares L Horn L Spigel DR Steins M Ready NE. Nivolumab versus docetaxel in advanced nonsquamous non-small-cell lung cancer. N Engl J Med (2015) 373(17):1627–39. doi: 10.1056/nejmoa1507643 PMC5705936 26412456

[40] Shu CA Gainor JF Awad MM Chiuzan C Grigg CM Pabani A. Neoadjuvant atezolizumab and chemotherapy in patients with resectable non-small-cell lung cancer: an open-label, multicentre, single-arm, phase 2 trial. Lancet Oncol (2020) 21(6):786–95. doi: 10.1016/s1470-2045(20)30140-6 32386568

[41] Munoz-Erazo L Rhodes JL Marion VC Kemp RA. Tertiary lymphoid structures in cancer - considerations for patient prognosis. Cell Mol Immunol (2020) 17(6):570–5. doi: 10.1038/s41423-020-0457-0 PMC7264315 32415259

[42] Di Caro G Bergomas F Grizzi F Doni A Bianchi P Malesci A. Occurrence of tertiary lymphoid tissue is associated with T-cell infiltration and predicts better prognosis in early-stage colorectal cancers. Clin Cancer Res (2014) 20(8):2147–58. doi: 10.1158/1078-0432.ccr-13-2590 24523438

[43] Kroeger DR Milne K Nelson BH. Tumor infiltrating plasma cells are associated with tertiary lymphoid structures, cytolytic T cell responses, and superior prognosis in ovarian cancer. Clin Cancer Res (2016) 22(12):3005–15. doi: 10.1158/1078-0432.ccr-15-2762 26763251

[44] Gunderson A J Rajamanickam V Bui C Bernard B Pucilowska J Ballesteros-Merino C. Germinal center reactions in tertiary lymphoid structures associate with neoantigen burden, humoral immunity and long-term survivorship in pancreatic cancer. OncoImmunology (2021) 10(1):1900635. doi: 10.1080/2162402x.2021.1900635 33796412 PMC7993148

[45] He M He Q Cai X Liu J Deng H Li F. Intratumoral tertiary lymphoid structure (TLS) maturation is influenced by draining lymph nodes of lung cancer. J ImmunoTher Cancer (2023) 11(4). doi: 10.1136/jitc-2022-005539 PMC10124324 37072348

