# Supplementary material for: Low-dose radiotherapy promotes the formation of tertiary lymphoid structures in lung adenocarcinoma
Source: Front Immunol. 2024 Jan 8;14:1334408. doi: 10.3389/fimmu.2023.1334408 (PMC10800908; doi:10.3389/fimmu.2023.1334408)
Supplement: Supplementary file 1 [file DataSheet_1.docx]

Supplementary Material

## Supplementary Figure S1


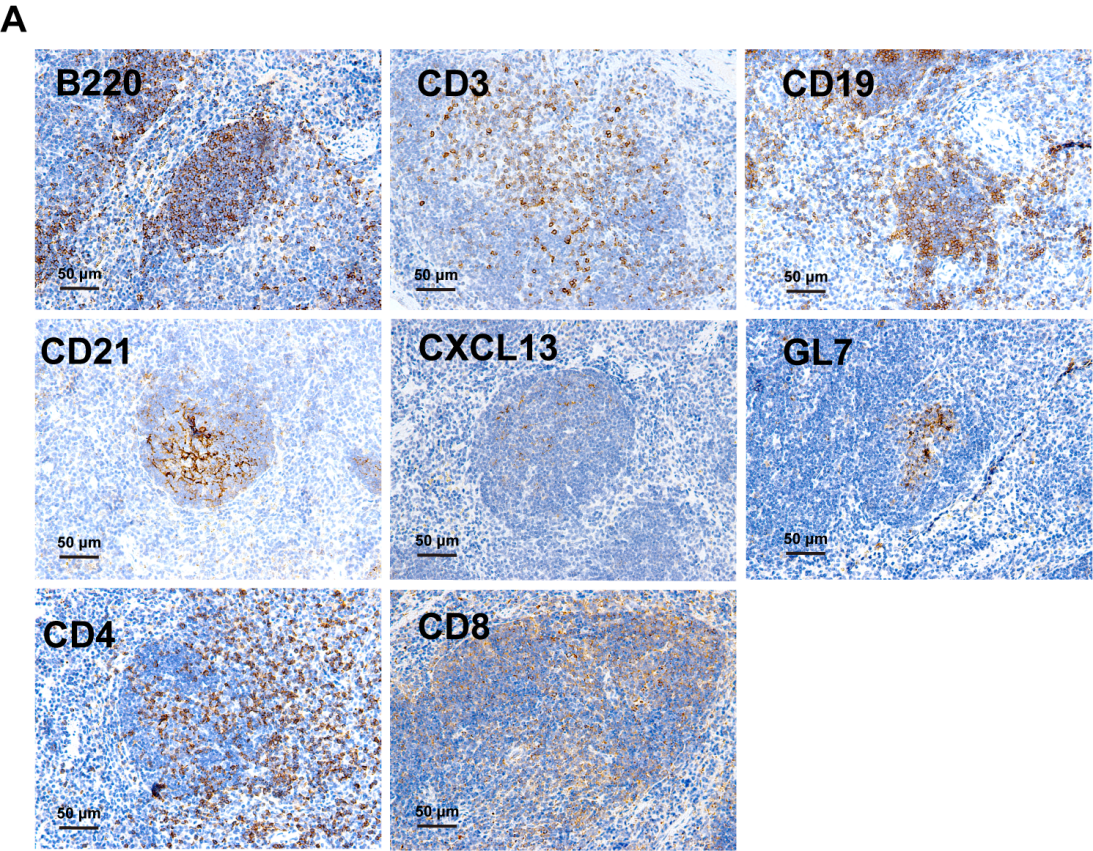


**Supplementary Figure S1.** A. Mouse spleen B220, CD3, CD19, CD21, CXCL13, GL7, CD4, CD8 IHC staining.
