# Supplementary material for: Low-dose radiotherapy promotes the formation of tertiary lymphoid structures in lung adenocarcinoma
Source: Front Immunol. 2024 Jan 8;14:1334408. doi: 10.3389/fimmu.2023.1334408 (PMC10800908; doi:10.3389/fimmu.2023.1334408)
Supplement: Supplementary file 2 [file DataSheet_2.docx]

Supplementary Material

## Supplementary Figure S2


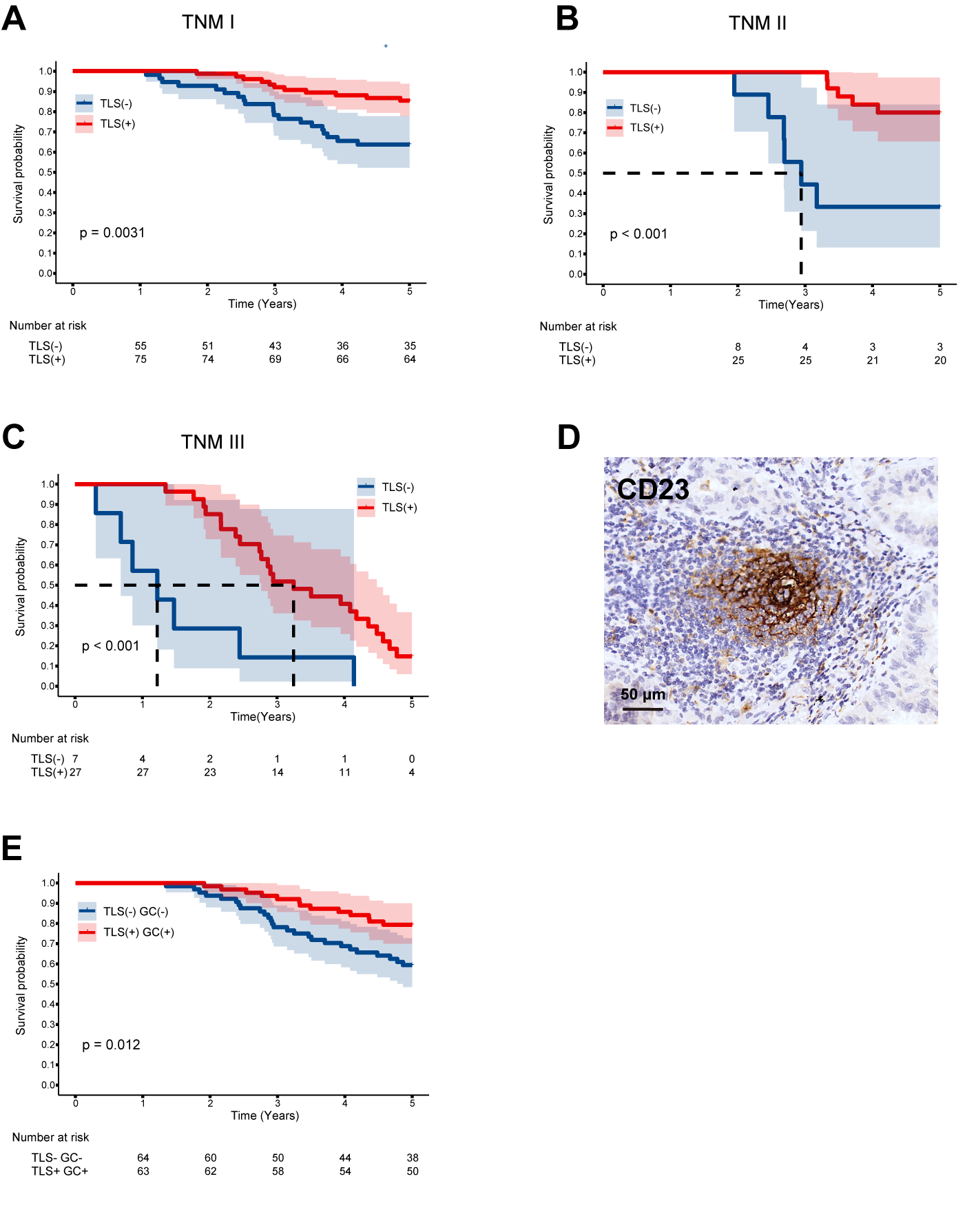


**Supplementary Figure S2.** A-C. Five-year Kaplan-Meier survival curves of TNM I, II and III in patients with and without intratumoral TLS. D.GC (CD23) IHC staining. E. five-year Kaplan-Meier survival curve of GC-/+ patients. The log-rank (Mantel-Cox) test was applied to compare the survival data in Figure A-D. IHC, Immunohistochemical techniques; GC, germinal center; TLS, tertiary lymphoid structure.
