# Supplementary material for: Low-dose radiotherapy promotes the formation of tertiary lymphoid structures in lung adenocarcinoma
Source: Front Immunol. 2024 Jan 8;14:1334408. doi: 10.3389/fimmu.2023.1334408 (PMC10800908; doi:10.3389/fimmu.2023.1334408)
Supplement: Supplementary file 3 [file DataSheet_3.docx]

Supplementary Material

## Supplementary Figure S3


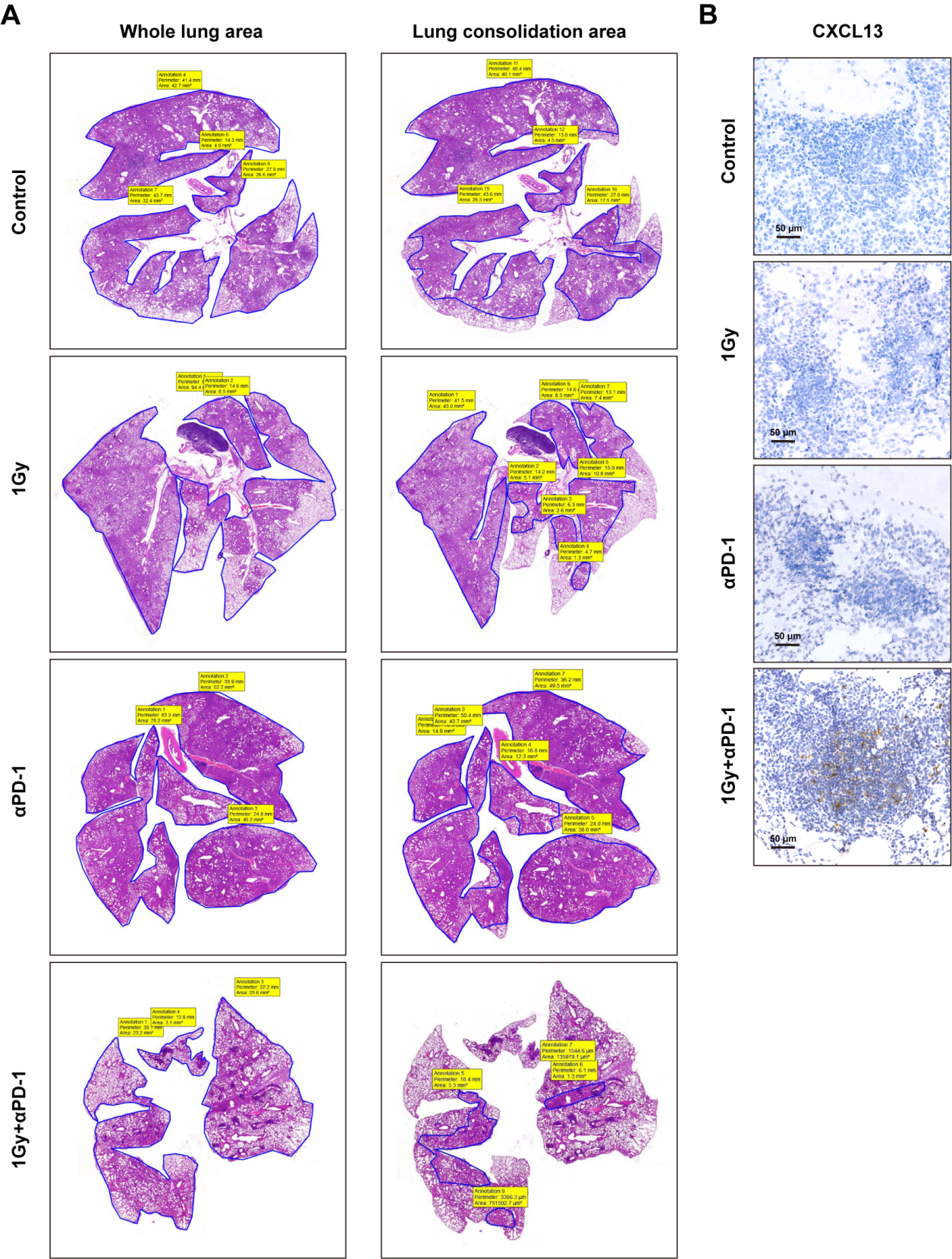


**Supplementary Figure S3.** A. Schematic diagram of lung section area and consolidation area. B. CXCL13 IHC staining in different treatment groups. IHC, immunohistochemical technique.
