# Supplementary material for: Low-dose radiotherapy promotes the formation of tertiary lymphoid structures in lung adenocarcinoma
Source: Front Immunol. 2024 Jan 8;14:1334408. doi: 10.3389/fimmu.2023.1334408 (PMC10800908; doi:10.3389/fimmu.2023.1334408)
Supplement: Supplementary file 4 [file Table_1.docx]

| Species Raised in | Antibody | Fluorochrome | Clone | Company | Catalog Number |
| --- | --- | --- | --- | --- | --- |
| Rat | CD45 | PECY5.5 | 30-F11 | eBioscience | 35-0451-82 |
| Armenian hamster | CD3 | PECY7 | 145-2C11 | eBioscience | 25-0031-82 |
| Rat | CD8a | APC | 54-6.7 | BioLegend | 100712 |
| Rat | CD19 | FITC | eBio1D3 (1D3) | eBioscience | 11-0193-82 |
| Rat | B220 | PE | RA3-6B2 | eBioscience | 14-0452-82 |

Supplementary Table S1. Antibodies for Flow Cytometry
